# Pregnancy-induced hypertension-related chorioretinitis resembling uveal effusion syndrome: A case report

**DOI:** 10.1097/MD.0000000000011572

**Published:** 2018-07-27

**Authors:** Tomohito Sato, Masaru Takeuchi

**Affiliations:** Department of Ophthalmology, National Defense Medical College, Saitama, Japan.

**Keywords:** pregnancy-induced hypertension, serous retinal detachment, uveal effusion syndrome

## Abstract

**Rationale:**

Pregnancy-induced hypertension (PIH) is a major cause of maternal and fetal mortality. Hypertensive choroidopathy is a preliminary sign of vasogenic edema in the choroid, and is associated with PIH. Here, we report a post-natal case of PIH-related chorioretinitis with bilateral severe serous retinal detachment (SRD) resembling uveal effusion syndrome.

**Patient concerns:**

A 35-year-old woman was diagnosed with severe PIH at 37 weeks of pregnancy. She underwent an emergency cesarean delivery. Four days after delivery, she perceived a sudden decrease of vision. At presentation, fundus examination demonstrated bullous SRD and multiple white mottles in the posterior poles of both eyes. Optical coherence tomography (OCT) showed macula edema and retinal pigment epithelium (RPE) folds. Indocyanine green angiography (ICGA) demonstrated delayed filling of choroidal circulation in the early phase and multiple hyperfluorescent spots in the mid phase.

**Diagnoses:**

PIH.

**Interventions:**

Antihypertension treatment alone resulted in gradual resolution of the SRD.

**Outcomes:**

At 463 days after delivery, fundus photographs of both eyes showed leopard spots corresponding to hyperautofluorescent spots with dark rim observed on fundus autofluorescence images.

**Lessons:**

Ophthalmologists should be aware of PIH-related chorioretinitis with similar clinical manifestations as uveal effusion syndrome, and should treat with antihypertensive agents in cooperation with obstetricians.

## 1 Introduction

Pregnancy-induced hypertension (PIH) is a complication resulting in maternal, fetal and newborn morbidity and mortality, in which there are central nervous system dysfunction, hepatocellular injury, thrombocytopenia, acute disseminated intravascular coagulation, oliguria, pulmonary edema, cerebrovascular events and placental abruption.^[1]^ In ophthalmology, PIH could induce acute retinal pigment epitheliopathy when malignant hypertension causes sudden choroidal ischemia.^[2]^ Hypertensive choroidopathy is a preliminary sign of vasogenic edema in the choroid, and is associated with PIH.^[3,4]^

Uveal effusion syndrome is a rare condition characterized by idiopathic spontaneous serous detachment of the retina and peripheral choroid, suggesting a primary scleral abnormality.^[5]^ Uveal effusion syndrome may be caused by a variety of disease states such as postoperative hypotony, scleral buckling procedures and scleritis.^[6]^ On the other hand, the syndrome may occur spontaneously with no apparent cause in one or both eyes of healthy individuals, particularly in middle-aged males.^[6]^

In this report, we present a case of PIH-related chorioretinitis with bilateral severe serous retinal detachment (SRD) resembling uveal effusion syndrome occurring after delivery, and discuss clinical features of the chorioretinitis.

## 2 Case report

**Figure 1:**
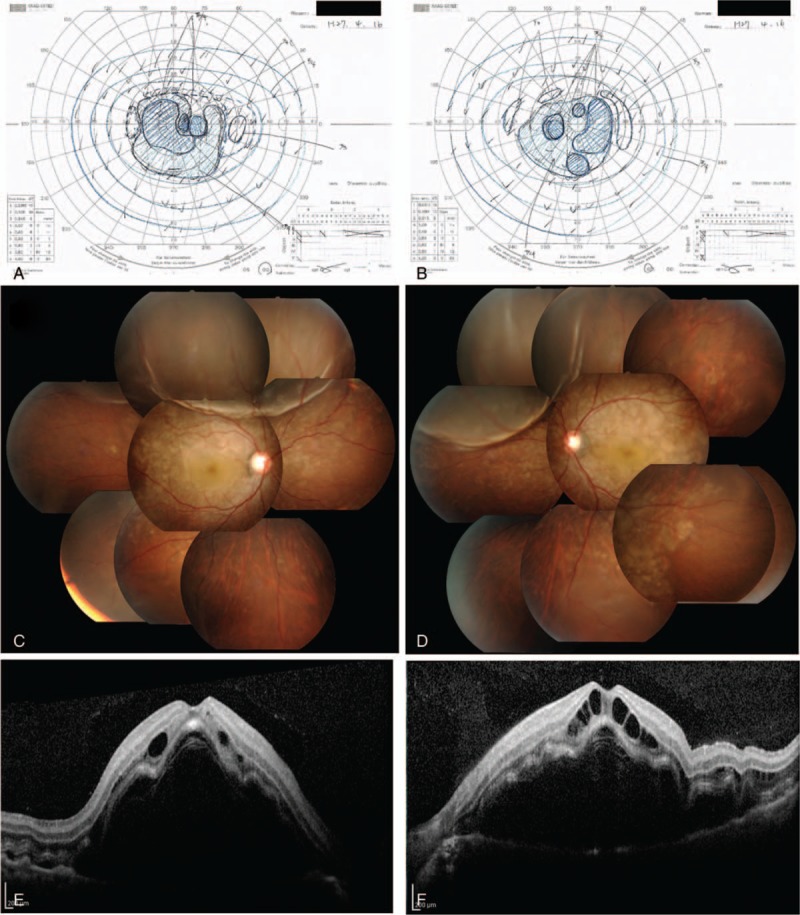
Visual field and fundus findings at 5 days after delivery. GP shows central scotomas over 20° in central visual field of the right eye (A) and left eye (B). Fundus images show bullous SRDs and multiple white mottles in the right eye (C) and left eye (D). OCT demonstrates macula edema and folds of the RPE layer in the right eye (E) and left eye (F). GP = Goldmann perimetry, OCT = optical coherence tomography, RPE = retinal pigment epithelium, SRD = serous retinal detachment.

**Figure 2:**
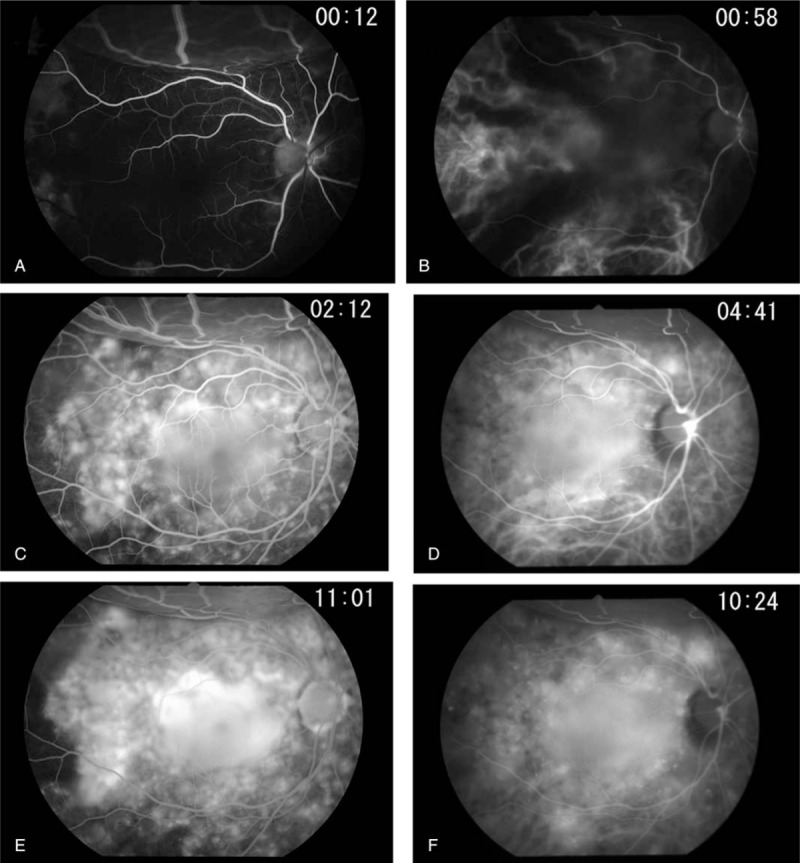
Angiographic findings of the right eye at 5 days after delivery. FA images show no delayed filling of retinal circulation in the early phase (A), multiple dye leakages in the mid phase (B) and poolings in the late phase (C). ICGA images demonstrate delayed filling of choroidal circulation in the early phase (D), multiple hyperfluorescent spots in the mid phase (E) and dye poolings in the late phase (F). FA = fluorescein angiography, ICGA = indocyanine green angiography.

**Figure 3:**
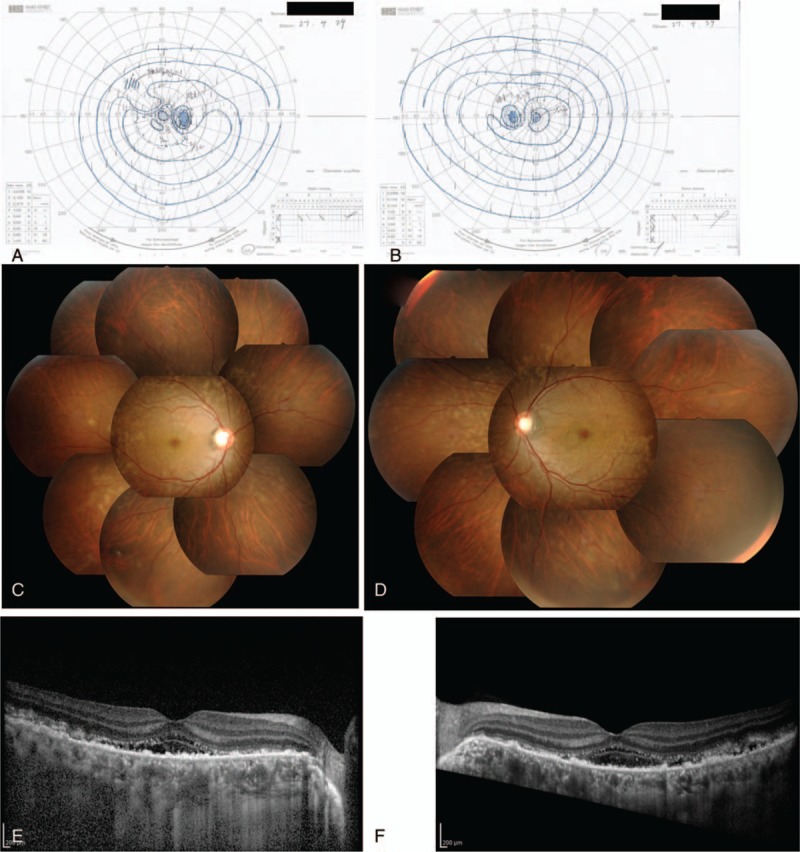
Visual field and fundus findings at 13 days after delivery. GP reveals central scotomas under 5° in central visual field, and expansion of Marriott's blind spots in the right eye (A) and left eye (B). Fundus photographs show resolution of bullous SRD, but persistence of multiple white mottles in the right eye (C) and left eye (D). OCT reveals choroidal thickening, alleviation of RPE folds, and multiple sub- and epi-RPE deposits with high brightness in the right eye (E) and left eye (F). GP = Goldmann perimetry, OCT = optical coherence tomography, RPE = retinal pigment epithelium, SRD = serous retinal detachment.

**Figure 4:**
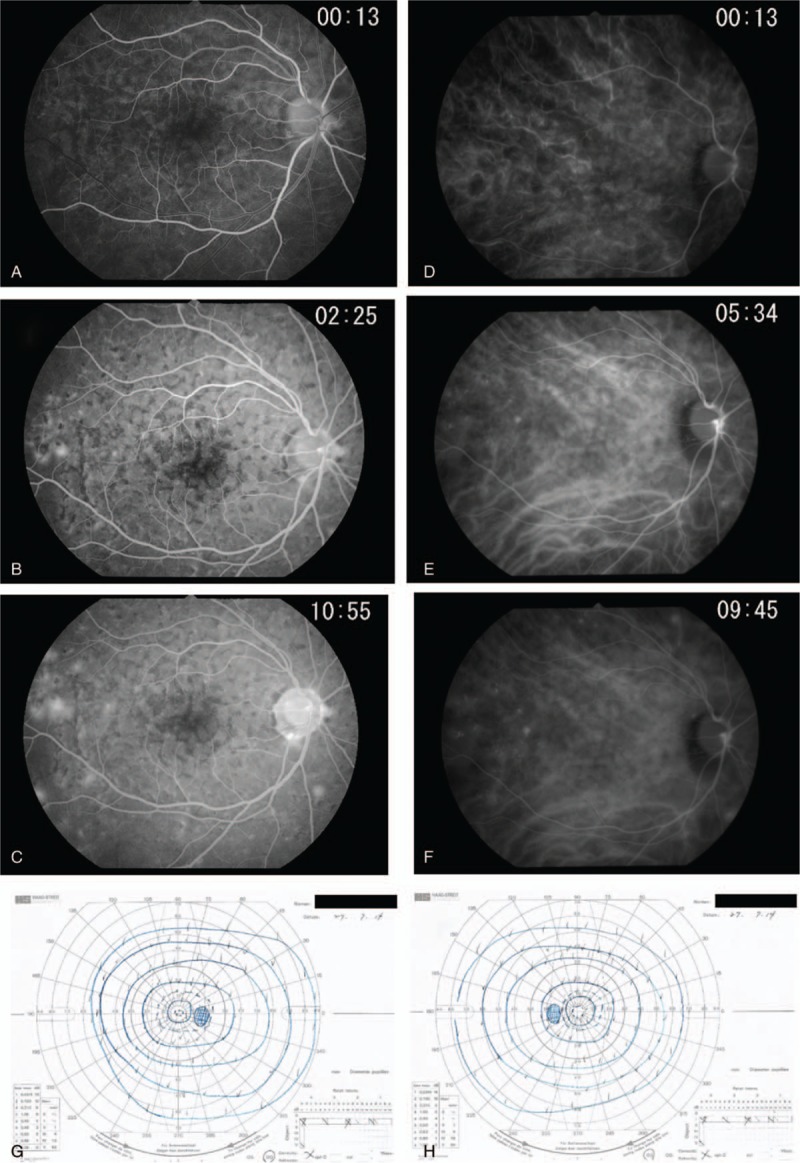
Angiographic findings of the right eye at 13 days after delivery, and visual field findings at 97 days after delivery. FA images show normal filling of retinal circulation in the early phase (A), multiple hypofluorescent spots and a few hyperfluorescent spots in the mid phase (B) and dye poolings at the hyperfluorescent spots during the late phase (C). ICGA images demonstrate normal filling of choroidal circulation in the early phase (D), and multiple hypofluorescent spots corresponding to the hypofluorescent spots observed on FA in the mid phase (E) and late phase (F). Central scotomas and expansion of Marriott's blind spots were not detected by GP in the right eye (G) and left eye (H). FA = fluorescein angiography, GP = Goldmann perimetry, ICGA = indocyanine green angiography.

**Figure 5:**
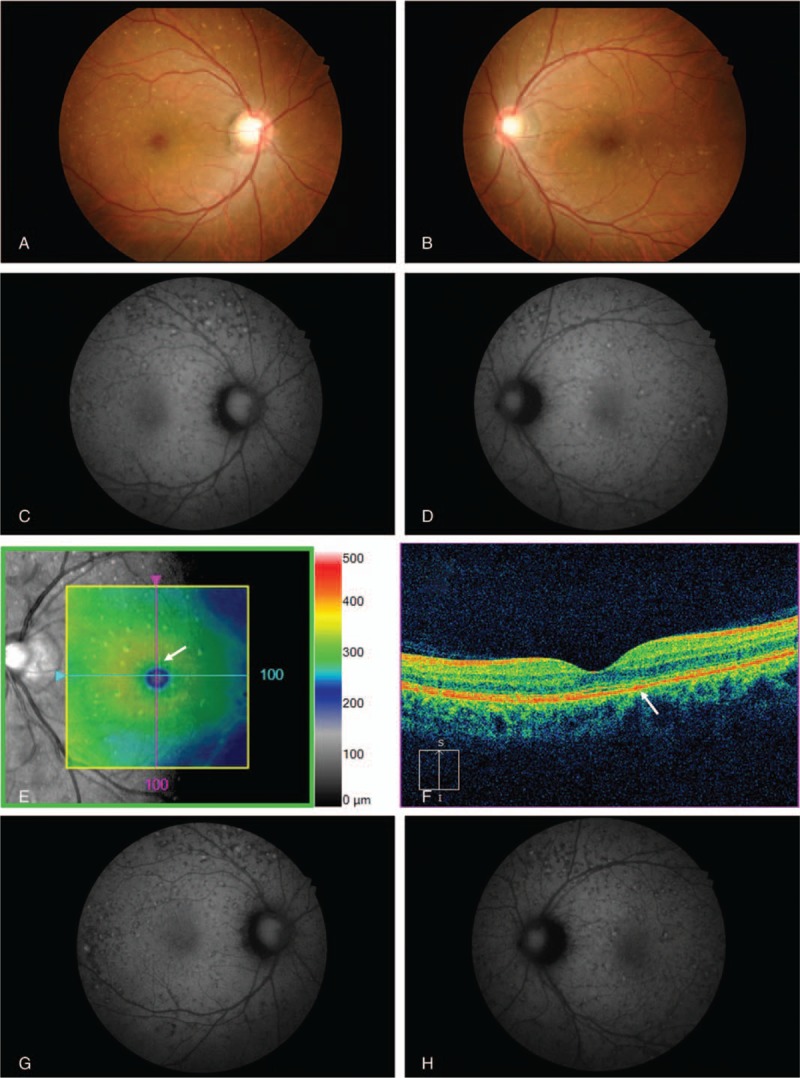
Fundus findings at 463 days and 3 years after delivery. At 463 days after delivery, fundus photographs demonstrate leopard spots in the right eye (A) and left eye (B). FAF images show hyperautofluorescent spots with dark rim corresponding to the leopard spots in the right eye (C) and left eye (D). OCT reveals focal deposits in the RPE layer corresponding to the hyperautofluorescent spots on FAF image in the left eye (E and D; white arrow). At 3 years after delivery, the leopard spot pattern with hyperautofluorescence spots on FAF images was attenuated but remained in the right eye (G) and left eye (H). FAF = fundus autofluorescence, OCT = optical coherence tomography, RPE = retinal pigment epithelium.

This study protocol was not approved by the Ethics Committee of National Defense Medical College as it was not deemed necessary, this being a retrospective case report. The Declaration of Helsinki was followed in this case report. Patient consent has been obtained for the publication of the contents in this report. A 35-year-old woman was diagnosed with severe PIH at 37 weeks of pregnancy, with blood pressure (BP) of 180/110 mm Hg.^[7]^ She underwent an emergency cesarean delivery. At the age of 29, she had undergone laser-assisted in-situ keratomileusis for correcting 7.0 D myopia in both eyes. Four days after delivery, she perceived a sudden decrease of vision. She had no nonspecific viral infection and no systemic symptoms such as headache, vertigo, and tinnitus before the sudden decrease of vision. At 5 days after delivery, she was referred to the National Defense Medical College Hospital, Japan. At presentation, her BP had decreased to 150/90 mm Hg, and her best-corrected visual acuity (BCVA) was 20/100 in both eyes. Critical fusion frequency (CFF) was 18 Hz in both eyes. Axial length was 24.83 mm in the right eye and 24.79 mm in the left eye. Goldmann perimetry (GP) demonstrated central scotomas over 20° in central visual fields of both eyes (Fig. 1A and B). Ophthalmoscopy showed no inflammatory cell in the anterior chamber and anterior vitreous cavity, but revealed apparent SRD and multiple white mottles in the posterior poles of both eyes (Fig. 1C and D). Spectral-domain optical coherence tomography (SD-OCT) (Heidelberg Engineering, Heidelberg, Germany, and Carl Zeiss Meditec AG, Jena, Germany) demonstrated macula edema and retinal pigment epithelial (RPE) folds (Fig. 1E and F). Fluorescein angiography (FA) showed multiple dye leakages in the mid phase (2 minutes after injection) (Fig. 2B) and multiple poolings in the late phase (11 minutes) (Fig. 2C). Indocyanine green angiography (ICGA) demonstrated delayed filling of the choroidal circulation in the early phase (58 seconds) (Fig. 2D), multiple hyperfluorescent spots in the mid phase (4 minutes) (Fig. 2E) and multiple poolings in the late phase (10 minutes) (Fig. 2F). The results of hematologic, urine, and cerebrospinal fluid (CSF) test were shown in Table 1, Table 2 and Table 3, respectively, and most of those were within normal limits. Aqueous humor levels of interleukin (IL)-6, interferon-inducible protein 10 and vascular endothelial growth factor were 14.3, 890.3 and 24.4 pg/mL, respectively (Table 4). Serum immunological tests were negative for human leukocyte antigen (HLA)-DR4, -DR53, -DQ4, and -DRβ1∗04. At 13 days after delivery, GP revealed alleviation of large central scotomas but persistence under 5° in central visual field, and expansion of Marriott's blind spots in both eyes (Fig. 3A and B). Funduscopy showed that the bullous SRDs were resolved, and multiple white mottles were attenuated but remained in both eyes (Fig. 3C and D). SD-OCT revealed choroidal thickening, alleviation of the RPE folds and multiple sub- and epi-RPE deposits with high brightness (Fig. 3E and F). FA showed multiple hypofluorescent spots and a few hyperfluorescent spots in the mid-phase (2 minutes) (Fig. 4B) and dye poolings at the hyperfluorescent spots during the late phase (10 minutes) (Fig. 4C). On ICGA images, delayed filling of the choroidal circulation in the early phase (13 seconds) was resolved (Fig. 4D), but multiple hypofluorescent spots remained, which corresponded to the hypofluorescent spots observed on FA in the late phase (9 minutes) (Fig. 4F). The SRDs gradually disappeared with only antihypertension treatment. Based on laboratory data and clinical course, PIH-related chorioretinitis but not Vogt–Koyanagi–Harada (VKH) disease was diagnosed as the cause of bilateral SRD. At 42 days after delivery, the SRDs in both eyes completely vanished. At 97 days after delivery, central scotomas and expansion of Marriott's blind spots were not observed by GP in both eyes (Fig. 4G and H). At 189 days after delivery, her BCVA recovered to be 20/20 in both eyes. CFF was 42 Hz in both eyes. At 463 days after delivery, fundus photographs demonstrated leopard spots in both eyes (Fig. 5A and B), which corresponded to hyperautofluorescent spots with dark rim on fundus autofluorescence (FAF) images (Fig. 5C and D). SD-OCT revealed focal deposits in the RPE layer corresponding to the hyperautofluorescent spots on FAF images (Fig. 5 E and F). The leopard spot pattern with hyperautofluorescence on FAF images was attenuated but remained even after 3 years (Fig. 5G and H).

**Table 1:**
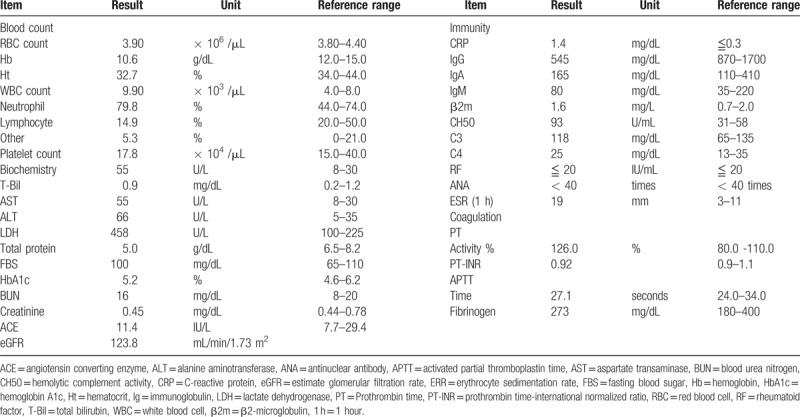
Hematologic data in the acute phase.

**Table 2:**
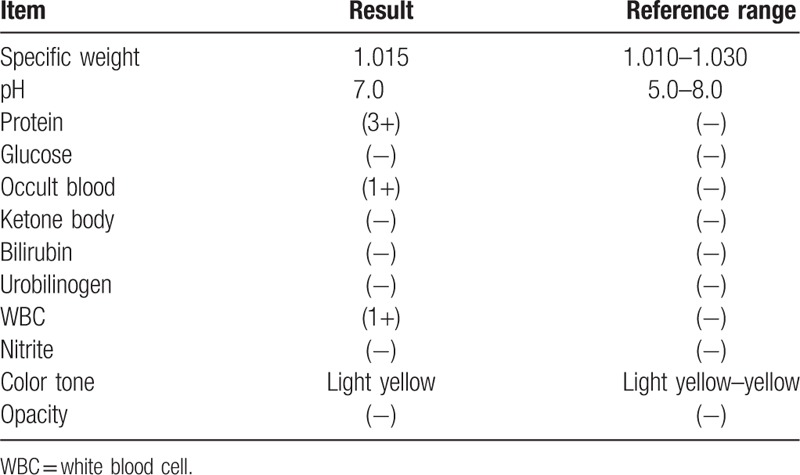
Urine data in the acute phase.

**Table 3:**
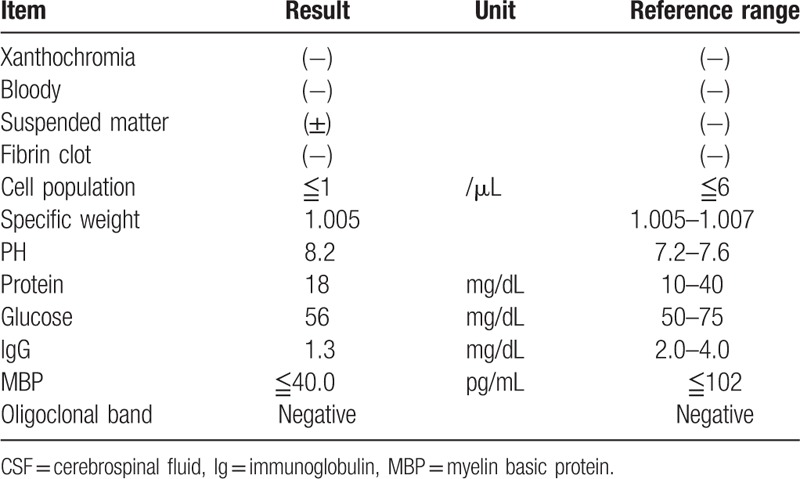
Cerebrospinal fluid data in the acute phase.

**Table 4:**
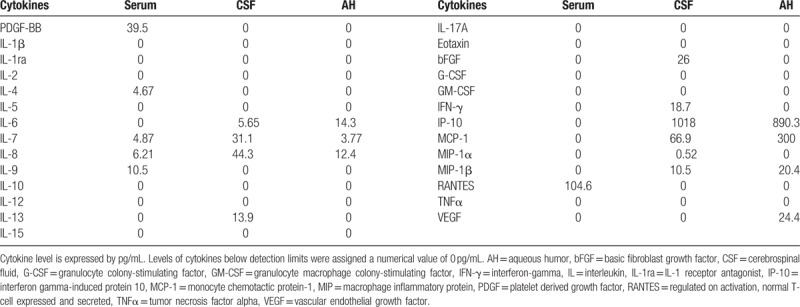
Serum, cerebrospinal fluid and aqueous humor levels of cytokines in the acute phase.

## 3 Discussion

Hypertensive disorders of pregnancy including pregnancy-induced hypertension (PIH) develop approximately 10% of all pregnant women in the world.^[1]^ PIH has been established as follows: systolic blood pressure (SBP) and diastolic blood pressure (DBP) are higher than 140/90 mm Hg.^[1,7]^ In ophthalmology, cortical blindness and central serous chorioretinopathy are known as PIH-related ocular diseases.^[8,9]^ However, secondary SRD after delivery is rare, and only a few papers described case reports of PIH patients with SRD, which developing after delivery.^[10–12]^

In monkey models, malignant hypertension induced choroidal fibrinoid necrosis, choriocapillaris nonperfusion, ischemic necrosis of RPE, compromise of the outer blood–retinal barrier and localized RPE detachment and/or SRD.^[13]^ Hypertensive choroidopathy is a manifestation of vasogenic edema of the choroid, and is associated with malignant hypertension, renal disease, pheochromocytoma and PIH.^[3,4]^ PIH-related retinochoroidal disorders could be divided into 3 types as follows: hypertensive retinopathy, characterized by retinal vascular occlusion with cotton wool patches, hypertensive choroidopathy (choroidal type), characterized by choroidal vascular occlusion with SRD, and mixed type, consisting of chorioretinal vascular occlusion.^[10,14]^ Our patient manifested bilateral bullous SRD but no cotton wool patches. SD-OCT revealed choroidal thickening and multiple RPE folds. Therefore, we consider that our patient developed the choroidal type of PIH rather than the retinal or combined type.

Uveal effusion syndrome has been recognized to be an abnormal condition, pooling fluid in the suprachoroidal space.^[15,16]^ Uveal effusion syndrome is classified into 3 subtypes based on the anatomical features as follows: type 1, an extreme small eye ball with highly hypermetropia, type 2, which a normal range size of eye ball with small ametropia, and type 3, a normal size eyeball. ^[17,18]^ Leopard spots by proliferation and migration of RPE cells are observe in uveal effusion syndrome,^[5]^ that are presented by multiple hypofluorescent spots on FA and ICGA.^[6,17,18]^ Furthermore, Okuda et al^[19]^ reported FAF images of leopard spots showing hyperautofluorescence in a patient with uveal effusion syndrome. In our patient, the eyeball size was not small but normal or rather large, and the findings of fundus, angiographic and FAF images were consistent with those of uveal effusion syndrome. Therefore, we speculate that the bilateral bullous SRD in this patient may have been induced by type 3 uveal effusion syndrome associated with choroidal type of PIH.

When blood–retinal barrier is impaired by inflammation, infection, neoplastic diseases, and retinal vascular occlusive diseases, SRD subsequently occurs.^[20]^ Several previous reports demonstrated post-natal SRD associated with PIH but the etiology remained unknown.^[10–12]^ The clinical features of SRD associated with PIH are similar to those observed in VKH disease.^[10]^ On the other hand, medical treatments of the 2 diseases are absolutely different. While the major treatment of PIH is BP management guided by obstetricians, VKH disease is generally treated with systemic and topical corticosteroids by ophthalmologists. At the acute phase of VKH disease, the clinical features are characterized by sudden blurred vision, bilateral posterior uveitis, diffuse granulomatous choroiditis with exudative retinal detachment, vitritis, and optic disc swelling.^[21]^ In VKH disease patients, inflammation and infiltration of inflammatory cells trigger changes of vascular permeability resulting in choroidal thickening, and RPE folds are observed over 70% of the eyes in these patients.^[22,23]^ ICGA images of VKH patients show characteristic multiple dark dots that are inflammatory granulomas with filling defects.^[24,25]^ Our patient had no prodromal symptoms, and ICGA showed no characteristic multiple dark dots. Furthermore, specific VKH disease-associated HLAs including HLA-B54, -DQ4, -DR4, -DR53 and -DRβ1∗04 were negative in this patient.^[25,26]^ Leukocytes in the CSF were almost absent, and was not consistent with VKH disease in the acute phase.^[27]^ The IL-6 level in the aqueous humor of our patient was considerably low compared with that of VKH disease patients.^[28]^ During the clinical course, she recovered promptly under only antihypertensive treatment. Therefore, although the clinical features of PIH-related chorioretinitis are apparently similar to those of VKH disease, the underlying pathophysiology of SRD is different from that of VKH disease.

In conclusion, PIH-related chorioretinitis is a rare disease and the major treatment of PIH is BP management guided by obstetricians. To select appropriate treatment option, ophthalmologists should be aware of PIH-related chorioretinitis with similar clinical manifestations of uveal effusion syndrome, and treat it with antihypertensive agents in cooperation with obstetricians.

## Author contributions

**Conceptualization:** Masaru Takeuchi.

**Data curation:** Tomohito Sato.

**Investigation:** Tomohito Sato.

**Resources:** Masaru Takeuchi.

**Writing – original draft:** Tomohito Sato.

**Writing – review & editing:** Masaru Takeuchi.

## References

[1] Kintiraki E Papakatsika S Kotronis G Pregnancy-induced hypertension. Hormones 2015;14:211–23. 26158653 10.14310/horm.2002.1582

[2] Hayreh SS Servais GE Virdi PS Fundus lesions in malignant hypertension. Ophthalmology 1986;93:45–59. 3951816 10.1016/s0161-6420(86)33775-8

[3] Tso MO Jampol LM Pathophysiology of hypertensive retinopathy. Ophthalmology 1982;89:1132–45. 7155524 10.1016/s0161-6420(82)34663-1

[4] Kinyoun JL Kalina RE Visual loss from choroidal ischemia. Am J Ophthalmol 1986;101:650–6. 3717247 10.1016/0002-9394(86)90764-6

[5] Forrester JV Lee WR Kerr PR The uveal effusion syndrome and trans-scleral flow. Eye (Lond) 1990;4:354–65. 2379646 10.1038/eye.1990.48

[6] Gass JD Uveal effusion syndrome. A new hypothesis concerning pathogenesis and technique of surgical treatment. Retina 1983;3:159–63. 6635350

[7] Visintin C Mugglestone MA Almerie MQ Management of hypertensive disorders during pregnancy: summary of NICE guidance. BMJ 2010;341:c2207. 20739360 10.1136/bmj.c2207

[8] Dinn RB Harris A Marcus PS Ocular changes in pregnancy. Obstet Gynecol Surv 2003;58:137–44. 12555046 10.1097/01.OGX.0000047741.79433.52

[9] Schultz KL Birnbaum AD Goldstein DA Ocular disease in pregnancy. Curr Opin Ophthalmol 2005;16:308–14. 16175045 10.1097/01.icu.0000179803.42218.cc

[10] Aoyagi R Hayashi T Tsuneoka H Choroidal thickening and macular serous retinal detachment in pregnancy-induced hypertension. Int Med Case Rep J 2015;8:291–4. 26635487 10.2147/IMCRJ.S95442 PMC4646594

[11] Song YS Kinouchi R Ishiko S Hypertensive choroidopathy with eclampsia viewed on spectral-domain optical coherence tomography. Graefes Arch Clin Exp Ophthalmol 2013;251:2647–50. 24013580 10.1007/s00417-013-2462-9

[12] Dewilde E Huygens M Cools G Hypertensive choroidopathy in pre-eclampsia: two consecutive cases. Ophthalmic Surg Lasers Imaging Retina 2014;45:343–6. 24972182 10.3928/23258160-20140617-02

[13] Kishi S Tso MO Hayreh SS Fundus lesions in malignant hypertension: I. A pathologic study of experimental hypertensive choroidopathy. Arch Ophthalmol 1985;103:1189–97. 4026650 10.1001/archopht.1985.01050080101029

[14] Saito Y Retinochoroidal changes in toxemia of pregnancy with the relation to hypertensive retinopathy and choroidopathy. Nippon Ganka Gakkai Zasshi 1990;94:748–55. 2239551

[15] Sakai H Yonahara M Sakai M Recurrent uveal effusion after laser iridotomy. Case Rep Ophthalmol 2017;8:26–30. 28203193 10.1159/000455038 PMC5301125

[16] Bellows AR Chylack LT Jr Hutchinson BT Choroidal detachment. Clinical manifestation, therapy and mechanism of formation. Ophthalmology 1981;88:1107–15. 7335316

[17] Uyama M Takahashi K Kozaki J Uveal effusion syndrome: clinical features, surgical treatment, histologic examination of the sclera, and pathophysiology. Ophthalmology 2000;107:441–9. 10711879 10.1016/s0161-6420(99)00141-4

[18] Gass JDM Jallow S Idiopathic serous detachment of the choroid, ciliary body, and retina (uveal effusion syndrome). Ophthalmology 1982;89:1018–32. 7177567 10.1016/s0161-6420(82)34685-0

[19] Okuda T Higashide T Wakabayashi Y Fundus autofluorescence and spectral-domain optical coherence tomography findings of leopard spots in nanophthalmic uveal effusion syndrome. Graefes Arch Clin Exp Ophthalmol 2010;248:1199–202. 20300765 10.1007/s00417-010-1352-7

[20] Amer R Nalci H Yalcindag N Exudative retinal detachment. Surv Ophthalmol 2017;62:723–69. 28506603 10.1016/j.survophthal.2017.05.001

[21] Attia S Khochtali S Kahloun R Vogt–Koyanagi–Harada disease. Expert Rev Ophthalmol 2012;7:565–85.

[22] Ishihara K Hangai M Kita M Acute Vogt–Koyanagi–Harada disease in enhanced spectral-domain optical coherence tomography. Ophthalmology 2009;116:1799–807. 19643489 10.1016/j.ophtha.2009.04.002

[23] Kato Y Yamamoto Y Tabuchi H Retinal pigment epithelium folds as a diagnostic finding of Vogt–Koyanagi–Harada disease. Jpn J Ophthalmol 2013;57:90–4. 23149670 10.1007/s10384-012-0212-x

[24] Herbort CP Mantovani A Bouchenaki N Indocyanine green angiography in Vogt–Koyanagi–Harada disease: angiographic signs and utility in patient follow-up. Int Ophthalmol 2007;27:173–82. 17457515 10.1007/s10792-007-9060-y

[25] Weisz JM Holland GN Roer LN Association between Vogt–Koyanagi–Harada syndrome and HLA-DR1 and -DR4 in Hispanic patients living in southern California. Ophthalmology 1995;102:1012–5. 9121744 10.1016/s0161-6420(95)30920-7

[26] Shi T Lv W Zhang L Association of HLA-DR4/HLA-DRB1∗04 with Vogt–Koyanagi–Harada disease: a systematic review and meta-analysis. Sci Rep 2014;4:6887. 25382027 10.1038/srep06887 PMC4225552

[27] Norose K Yano A Aosai F Immunologic analysis of cerebrospinal fluid lymphocytes in Vogt–Koyanagi–Harada disease. Invest Ophthalmol Vis Sci 1990;31:1210–6. 2142146

[28] Norose K Yano A Wang XC Dominance of activated T cells and interleukin-6 in aqueous humor in Vogt–Koyanagi–Harada disease. Invest Ophthalmol Vis Sci 1994;35:33–9. 8300361

